# Nosocomial transmission and outbreaks of coronavirus disease 2019: the need to protect both patients and healthcare workers

**DOI:** 10.1186/s13756-020-00875-7

**Published:** 2021-01-06

**Authors:** Mohamed Abbas, Tomás Robalo Nunes, Romain Martischang, Walter Zingg, Anne Iten, Didier Pittet, Stephan Harbarth

**Affiliations:** 1 Infection Control Programme, Geneva University Hospitals and Faculty of Medicine, Geneva, Switzerland; 2 Health Protection Research Unit, Imperial College London, London, UK; 3 Infectious Diseases Service, Hospital Garcia de Orta, EPE, Almada, Portugal; 4 University of Geneva, Geneva, Switzerland

**Keywords:** COVID-19, SARS-CoV-2, Infection prevention and control, Healthcare-associated infection, Nosocomial outbreaks

## Abstract

**Objectives:**

To compile current published reports on nosocomial outbreaks of severe acute respiratory syndrome coronavirus 2 (SARS-CoV-2), evaluate the role of healthcare workers (HCWs) in transmission, and evaluate outbreak management practices.

**Methods:**

Narrative literature review.

**Short conclusion:**

The coronavirus disease 2019 (COVID-19) pandemic has placed a large burden on hospitals and healthcare providers worldwide, which increases the risk of nosocomial transmission and outbreaks to “non-COVID” patients or residents, who represent the highest-risk population in terms of mortality, as well as HCWs. To date, there are several reports on nosocomial outbreaks of SARS-CoV-2, and although the attack rate is variable, it can be as high as 60%, with high mortality. There is currently little evidence on transmission dynamics, particularly using genomic sequencing, and the role of HCWs in initiating or amplifying nosocomial outbreaks is not elucidated. There has been a paradigm shift in management practices of viral respiratory outbreaks, that includes widespread testing of patients (or residents) and HCWs, including asymptomatic individuals. These expanded testing criteria appear to be crucial in identifying and controlling outbreaks.

## Introduction

Severe acute respiratory syndrome coronavirus 2 (SARS-CoV-2), first described in December 2019, causes coronavirus disease 2019 (COVID-19), and has been declared as a Public Health Emergency of International Concern by World Health Organization (WHO) on 30 January 2020. The number of COVID-19 patients requiring hospitalisation has been high, although data on proportions of cases requiring hospitalisation are not only scarce, but also difficult to compare due to different testing strategies and possibly hospital admission criteria. For example, the cumulative numbers of hospitalised cases (and proportions among documented cases) are 4036 (12.7%) in Switzerland (30 June 2020) [1], 126,695 (42.0%) in England (16 June 2020) [2], and 103,451 (64.1%) in France (24 June 2020) [3].

The high numbers of hospitalised patients placed a large burden on healthcare systems, which have had to adapt their surge capacity and infrastructure [4, 5]. Hospitals admitting COVID-19 patients have practised cohorting in accordance with recommendations from infection prevention and control (IPC) professional societies [6–9]. The resulting hospital-wide colonisation pressure of SARS-CoV-2 is thus high, potentially exposing healthcare workers (HCWs) and non-COVID-19 patients to nosocomial SARS-CoV-2 acquisition and transmission.

Furthermore, because HCWs are at the interface between healthcare settings and the community, where there is significant transmission, combined with the fact that as essential workers they are not confined, they may also play a role in initiating or amplifying outbreaks in settings such as hospitals [10, 11] and long-term care facilities (LTCFs) [12].

The aim of this narrative review is to provide an overview of the existing literature regarding SARS-CoV-2 transmission and outbreaks in healthcare settings, to evaluate the role of HCWs in these outbreaks, and to highlight key IPC practices in outbreak management and prevention.

## Methods

A narrative literature review was performed using the PubMed and Google Scholar databases up to July 22, 2020, searching for current published reports on nosocomial outbreaks of SARS-CoV-2. Publications evaluating the role of healthcare workers (HCWs) in transmission and evaluating outbreak management practices were analysed. In Table 1, a summary of healthcare-associated outbreaks of SARS-CoV-2 involving patients, long-term care facility residents, and HCWs is presented.

**Table 1:** Summary of healthcare-associated outbreaks of SARS-CoV-2 involving patients, long-term care facility residents, and healthcare workers

Country Author name	Study design Setting	Source population	Risk factors for infection analysed	Attack rate (%)	Infection control measures	References
*China*						
Li et al.	Outbreak investigation Hospital	205 patients 148 HCWs	Not performed	6.3 (patients) 8.1 (HCWs)	“Comprehensive protective measures such as quarantine and disinfection”	[82]
Wang et al.	Outbreak investigation Hospital	27 HCWs 10 relative	Not performed	NR	Isolation of infected HCWs	[32]
*Hong Kong Special Administrative Region*						
Cheng et al.	Suspected hospital outbreak (“near miss”)	413 HCWs	Not performed	0.0	Reduction of bed occupancy Active surveillance of hospitalised patients Linking contact tracing to the hospital Visitor restriction Cohorting in AIIRs Segregation of staff working in high-risk areas	[64]
Wong et al.	Suspected hospital outbreak (“near miss”)	71 staff 49 patients	N/A	0.0 (staff) 0.0 (patients)	Quarantine of close contacts Symptom screening and monitoring Universal masking Enhanced environmental cleaning	[20]
*France*						
Vanhems et al.	Outbreak investigation Hospital	35 patients NR staff	Not performed	20.0 (patients) NR (staff)	“Strict infection control measures” “Close monitoring of suspected cases”	[83]
Luong-Nguyen et al.	Outbreak investigation Hospital	301 patients	Not performed	4.9 (patients)	Reduction of bed occupancy Screening patients on admission Visitor restrictions Universal masking Cohorting	[61]
*Germany*						
Kabesch et al.	Outbreak investigation Hospital	562 staff	Not performed	5.2	Symptom monitoring of close contacts Universal masking Physical distancing in non-clinical areas	[62]
Schneider et al.	Hospital-based surveillance	66 HCWs	N/A	0.0	Ban on elective surgery (incl. outpatient clinics) Visitor restrictions Universal masking Screening prior to transfer in rehabilitation	[63]
Schwierzeck et al.	Outbreak investigation Dialysis unit	28 HCWs (8 lab confirmed) 13 patients (3 lab confirmed) 7 relatives (1 lab confirmed)	Exposure analysis only	NR	Quarantine of exposed HCWs (or work with surgical mask if asymptomatic) Symptom monitoring Expanded testing criteria	[47]
*Singapore*						
Wee et al.	Outbreak investigation Hospital	14 HCWs	Not performed	NR	Self-isolation of HCWs if symptomatic or in close contact Symptom monitoring Syndromic surveillance of HCWs (staff clinic)	[84]
Ng et al.	Suspected hospital outbreak (“near miss”)	41 HCWs	Not performed	0.0	Self-isolation of HCWs in close contact Symptom monitoring Expanded testing criteria	[48]
*South Africa*						
Lessels et al.	Outbreak investigation Hospital	80 staff 39 inpatients	Not performed	4.0 (staff) NR (patients)	Surveillance or self-monitoring of exposed contacts (HCWs & patients) Quarantine of selected HCWs Cohorting Cancellation of elective surgical procedures Expanded testing criteria (all staff) Enhanced environmental cleaning Hospital closure	[49]
*United Kingdom*						
Graham et al.	Outbreak investigation Nursing homes (n = 4)	70 staff 394 residents	Not performed	4.3 (staff) 32.0 (residents)	Self-isolation of HCWs if symptomatic or in close contact Expanded testing criteria (serial testing) Cohort wards	[39]
Rickman et al.	Outbreak investigation Hospital	435 patients	Not performed	NR (15.0% of all COVID-19 cases)	Cohort wards Visitor restrictions Expanded testing criteria Cohorting suspected cases Staff screening Universal masking	[50]
*United States*						
Arons et al.	Outbreak investigation Nursing home	89 residents Staff data NR	Not performed	64.0 (residents) NR (staff)	Visitor restrictions Cancellation of communal activities Facility-wide transmission precautions (PPE use) Expanded testing criteria (serial prevalence) Cohorting?	[34]
Baker et al.	Outbreak investigation Hospital	1 index patient 44 HCWs	Not performed	4.7 (staff)	Universal masking Symptom monitoring Expanded testing criteria (for contacts)	[51]
Dora et al.	Outbreak investigation Nursing home	99 residents 136 staff	NR	19.2 (residents) 4.4 (staff)	Expanded testing criteria (serial prevalence) Symptom screening Cohorting Restricting staff movement across wards	[52]
Heinzerling et al.	Outbreak investigation Hospital	121 HCWs	Case–control study (COVID-19 vs. non-COVID-19 HCWs)	2.5	Self-isolation in case of close contact Symptom monitoring	[85]
McMichael et al.	Outbreak investigation Nursing home network	101 residents 50 HCWs 16 visitors	Not performed	NR	PPE training Hand hygiene assessments Audits of PPE use Reviews of environmental cleaning and disinfection practices Mandatory screening of HCWs Visitor restrictions Physical distancing Restricting resident movement and group activities	[40]
Kimball et al.	Outbreak investigation Nursing homes	82 residents	Not performed	28.0	Expanded testing criteria Universal isolation precautions Visitor restrictions Symptom monitoring (residents) Fever screening (HCWs)	[41]
Patel et al.	Outbreak investigation Nursing home	127 residents 112 staff		26.0 (residents) 17.0 (staff)	Expanded testing criteria Symptom screening (visitors, staff) Visitor restriction Universal masking Cohorting Enhanced training (hand hygiene, environmental cleaning, etc.)	[53]
Roxby et al.	Outbreak investigation Nursing homes	80 residents 62 staff members	Not performed	3.8 (residents) 3.2 (staff)	Physical distancing Visitor restrictions Restriction of communal activities Self-isolation of symptomatic HCWs Enhanced environmental cleaning Expanded testing criteria (serial testing)	[54]

*AIIR *airborne infection isolation room, *CDC* Centers for Disease Control and Prevention (US), *COVID-19* coronavirus disease 2019, *HAI* healthcare-associated infection, *HCW* healthcare worker, *NHC* National Health Commission (People’s Republic of China), *NR* not reported or performed, *PPE* personal protective equipment

### Risk of nosocomial transmission to and from healthcare workers

HCWs are at increased risk of SARS-CoV-2 exposure while caring for COVID-19 patients. However, precise epidemiologic data on SARS-CoV-2 transmission to HCWs are scarce to date, consisting essentially of case series or cross-sectional studies [14, 15], small cohort studies [16], reports from governmental agencies [17], or articles in the lay press [18]. Furthermore, problems with estimates are compounded by uncertain exposure histories and differential screening policies for HCWs [19]. A systematic review identified 15 studies in the scientific literature (including 7 non-peer-reviewed preprints) on burden of SARS-CoV-2 infection in HCWs up to 5 May 2020; however, many of the studies did not provide denominators of exposed HCWs, and the methodological quality was low [13]. Few studies reported on risk factors of SARS-CoV-2 acquisition by HCWs [16, 20].

Currently, attack rates of SARS-CoV-2 in HCWs are difficult to estimate. Data from the Italian *Istituto Superiore di Sanità* [17] suggested that, as of 6 May 2020, 23,718 HCWs were infected, representing 11.1% of all microbiologically confirmed cases in the country; however, it is unclear if these were community or healthcare-acquired infections and whether all these HCWs delivered direct care to COVID-19 patients, particularly in hospital settings. An earlier study from a Dutch hospital reported that within 2 weeks of the first Dutch case, 6% of symptomatic HCWs, representing 0.9% of the total workforce, were infected [21]. A prospective cohort study in an English hospital showed that the proportion of SARS-CoV-2-infected HCWs was relatively low (< 7%) and comparable to the prevalence in the community [22]. In another English hospital with a restrictive screening policy (i.e. only in presence of new continuous cough or fever), the proportion of SARS-CoV-2 positive HCWs was 20% [23]. According to the analysis by Xiang et al. of data from HCWs infected in China, many HCWs who were infected in the healthcare setting were early in the epidemic (i.e. before end of January 2020) and were probably due to either insufficient preparedness in terms of appropriate use of personnel protective equipment (PPE) or due to shortages in PPE [24].

It is also important to analyse COVID-19 related deaths in HCWs. In fact, a government report from England and Wales showed that age-adjusted COVID-19 mortality for HCWs was 10.2 deaths per 100,000 males (n = 43 deaths) and 4.8 deaths per 100,000 females (n = 63 deaths); this was not found to be different from mortality in the general working population [25].

**Fig. 1:**
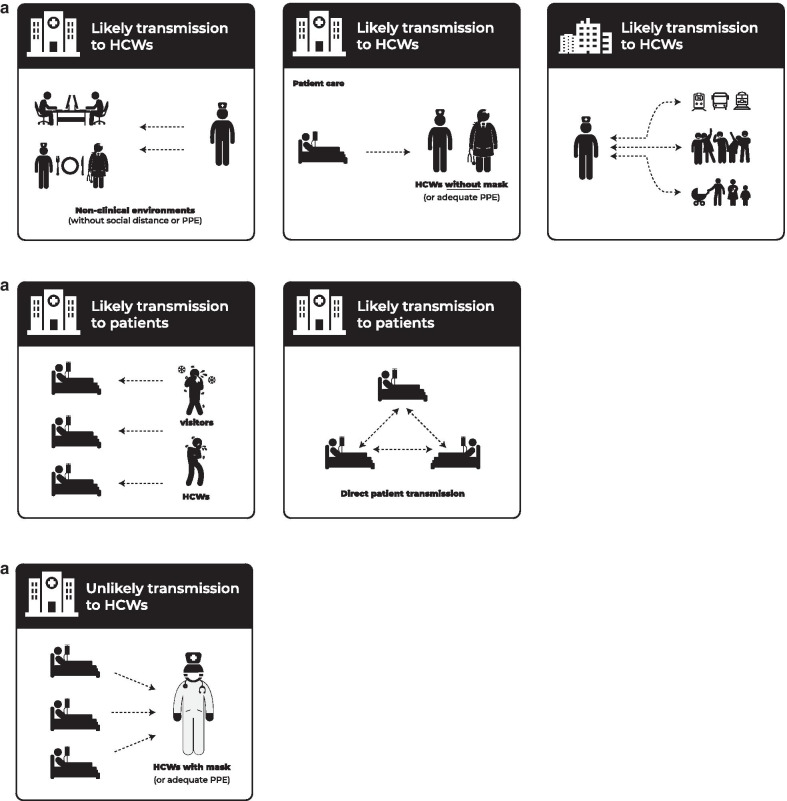
Transmission pathways of SARS-CoV-2. **a** Likely modes of SARS-CoV-2 transmission to HCWs in healthcare environments (both direct patient care and non-clinical settings) and in the community. **b** Likely modes of SARS-CoV-2 transmission to patients in healthcare settings. **c** Unlikely modes of SARS-CoV-2 transmission to HCWs in healthcare environments

It remains unclear, particularly in settings with extensive community transmission prior to lockdown events, to what extent HCWs were infected in the community or during professional activity (Fig. 1) [16]. For the latter, it is not known whether professional exposure to COVID-19 is limited to patient care or to cross-transmission between peers during activities other than patient care. Indeed, as HCWs are younger than the general population [26], and considering that the proportion of asymptomatic or pauci-symptomatic SARS-CoV-2 infections is inversely proportional to increasing age [27], it is plausible that transmission of SARS-CoV-2 to HCWs may occur outside work or during peer-to-peer interaction outside direct patient care. In fact, several studies using whole genome sequencing that suggest that HCWs can be infected in the community and possibly help amplify SARS-CoV-2 outbreaks in the healthcare setting. A study performed in three hospitals in the Netherlands, which combined epidemiological and genetic data, shows that widespread community transmission of SARS-CoV-2 and super-spreading events, such as carnivals, were probable sources of infection in some HCWs [11]. A study performed in 2 skilled nursing facilities in Minnesota, also using epidemiological end genetic information, suggested cross-transmission within the facility. However, some healthcare workers also presented genetically distinct strains, probably acquired in the community setting [12].

A large hospital-wide screening programme has shown that 30 of 61 (49%) SARS-CoV-2 positive HCWs were asymptomatic, though 40% of them were found to have had mild symptoms preceding a positive test (i.e. were pauci-symptomatic) [28]. A pre-pandemic survey suggested that 56% of HCWs with any influenza-like symptoms would go to work; the proportion of those with “mild” symptoms (e.g. sore throat, nasal discharge, etc.) who would still go to work is higher (89–99%) [29]. This also seems true for COVID-19; a report of HCWs screened during an outbreak in a LTCF in the US showed that 64.6% of HCWs with confirmed SARS-CoV-2 infection worked while they were symptomatic, and for a median duration of 2 days (range 1–10) [30]. This clearly indicates a high risk of HCWs being vectors of healthcare-associated viral respiratory illness, such as COVID-19, when caring for uninfected patients [31].

A report of a nosocomial outbreak involving 25 HCWs with microbiologically-documented SARS-CoV-2 infection spanning from January to February 2020 in Wuhan, China, was published in June [32]. The outbreak started in the Department of Neurosurgery of a hospital in Wuhan where HCWs (n = 12) were managing 2 index patients with documented SARS-CoV-2 infection (unclear if healthcare-associated). HCWs of other departments (n = 13) were subsequently infected, as were 10 of 43 family members of all HCWs. A detailed contact tracing investigation identified 9 transmission clusters, with 2 index patients infecting 5 HCWs with reasonable probability, and 2 suspected transmission events. These HCWs were involved in probable transmission to 1 other HCW and suspected transmission to 6 additional HCWs. Genome sequencing was performed on samples obtained from 11 HCWs and 1 family member; the phylogenetic tree showed 4 clades, with one clade involving 5 HCWs and a family member. This detailed report clearly suggests that HCWs can transmit SARS-CoV-2 to other HCWs. Unfortunately, the sequences of the index patients were not available, and therefore the first step in the presumed transmission chain could not be analysed genetically.

In summary, since HCWs are at the interface between hospitals and LTCFs on the one hand and the community on the other, they may serve as reservoirs, vectors, or victims of SARS-CoV-2 cross-transmission.

### Definitions of healthcare-associated COVID-19 in hospitalized patients

Currently, there are no universally accepted definitions of healthcare-associated COVID-19. Table 2 summarises a number of official published definitions of healthcare-associated COVID-19. Considering the uncertainty revolving around atypical symptoms (or lack thereof), incubation periods, transmission in the pre-symptomatic stages of infection, and the controversy over asymptomatic infection, this is hardly surprising. This is also compounded by the fact that admission-based screening practices vary by epidemiology (mid-wave vs. post-wave), place (LTCF vs. acute hospital ward), availability of testing, and country. Nonetheless, a balance should be sought between a sensitive or specific case definition that is applicable for outbreak management. Conservative definitions (e.g. the 48-h post-admission rule, usually applied for healthcare-associated infections) are likely to be more sensitive in detecting healthcare-associated COVID-19 for operational purposes, such as triggering outbreak responses. Yet, they will potentially overestimate the true number of healthcare-associated COVID-19, as the median incubation period until the development of COVID-19 symptoms is approximately 5 days [33]. Therefore, by using the 48-h post-admission rule, patients with probable community acquired infections will still be included in this definition.

**Table 2:** Official definitions of healthcare-associated coronavirus disease 2019 (COVID-19) cases and outbreaks

Country	Definition of cases	Definition of outbreak	References
England	Probable healthcare-associated COVID-19: a single inpatient who develops COVID-19 more than 7 days after hospital admission	Two or more cases in a single setting are detected that have become symptomatic or detected on screening on or after day eight of hospital admission	[86]
Canada	Not reported	LTCFs: a single laboratory-confirmed case of COVID-19 in a staff member (or resident)	[87]
Ireland	• Onset of compatible symptoms ≥ 7 days after admission • Onset of compatible symptoms 3–6 after admission if epidemiologically linked to hospital exposure • Onset of clinical features of COVID-19 on day 1 or 2 after admission are considered community acquired unless epidemiologically linked to hospital exposure during a recent hospital admission • If onset of clinical features cannot be defined, a case by case assessment is required taking account of the date of sampling relative to the date of admission, the CT value of the test result and epidemiological evidence of a link to hospital exposure.	Not reported	[88]
Switzerland	• Patient with new onset of COVID-19 compatible signs and symptoms* at least 5 days after hospital admission and a positive PCR result and/or thorax CT scan suggestive of COVID-19 • For hospitals with universal admission screening: Patient with negative PCR on admission and new onset of COVID-19 compatible symptoms and/or a positive PCR result at least 5 days after hospital admission	Detection of ≥ 3 nosocomial COVID-19 cases with a possible epidemiological (temporal and local) link	[89]
United States	• NOT considered nursing home onset COVID-19: • Residents who were known to have COVID-19 on admission to the facility and were placed into appropriate Transmission-Based Precautions to prevent transmission to others in the facility. • Residents who were placed into Transmission-Based Precautions on admission and developed SARS-CoV-2 infection within 14 days after admission	Notification required in case of: Residents or HCP with suspected or confirmed COVID-19 Residents with severe respiratory infection resulting in hospitalization or death ≥ 3 residents or HCP with new-onset respiratory symptoms within 72 h of each other	[90, 91]
European Centre for Disease Prevention and Control	• Community-associated COVID-19 (CA-COVID-19): Symptoms present on admission or with onset on day 1 or 2 after admission • Symptom onset on days 3-7 and a strong suspicion of community transmission.. • Indeterminate association (IA-COVID-19): Symptom onset on day 3-7 after admission, with insufficient information on the source of infection to assign to another category. • Probable healthcare-associated COVID-19 (HA-COVID-19): Symptoms onset on day 8-14 after admission • Symptom onset on day 3-7 and a strong suspicion of healthcare transmission. • Definite HA-COVID-19: Symptom onset on day ≥14 after admission	Not provided	https://www.ecdc.europa.eu/en/covid-19/surveillance/surveillance-definitions. Accessed December 26, 2020

### Healthcare-associated transmission of SARS-CoV-2 in hospitalized patients or residents

The fact that HCWs can acquire SARS-CoV-2 in the community, combined with the practice that many hospitals admit both COVID-19 and non-COVID-19 patients at the same sites, produces a high colonisation pressure of SARS-CoV-2 in hospital settings, exposing both susceptible patients and HCWs to the risk of healthcare-associated SARS-CoV-2 infection. Although current recommendations highlight the importance of cohorting COVID-19 patients, truly isolating them from susceptible non-COVID-19 patients is not always possible. In fact, the long incubation period of SARS-CoV-2 infection and the high proportion of asymptomatic/pauci symptomatic infections, even in LTCFs [34], creates the perfect environment for silent transmission in the healthcare setting. In particular, elderly patients who are hospitalised in geriatric wards, or transferred to rehabilitation clinics, as well as those in LTCFs, are frail and have comorbidities making them more vulnerable to complications resulting from SARS-CoV-2 infections [35]. Moreover, the longer duration of hospital stay increases the risk of healthcare-acquired COVID-19. Indeed, during the pandemic, many hospitals restricted or suppressed visits, meaning that healthcare-associated SARS-CoV-2 infections were almost exclusively due to patient-to-patient or HCW-to-patient transmission.

Outbreaks in LTCFs are a major public health concern, yet few countries report data. In England, data from the government show that up to 21 June 2020, there were 6,538 SARS-CoV-2 outbreaks in a total of 15,507 facilities [36]. In France, there were 3,375 clusters (≥ 1 case) in nursing homes, out of a total of 8,158 reports that involved 34,283 COVID-19 cases [3]. In the canton of Geneva, the region of Switzerland with the highest burden of COVID-19, 40.3% of all deaths were in nursing homes [37]. In Belgium, 8.7% of all confirmed COVID-19 cases were in nursing home residents [38]. To date, there are few published reports of healthcare-associated outbreaks (Table 1), suggesting that this is a sensitive issue and that there may be some degree of publication bias.

An outbreak of SARS-CoV-2 across 4 nursing homes in London was reported involving 394 residents and 70 staff [39]. Extensive testing, including on asymptomatic residents, showed a high attack rate in residents (32.0%), with high mortality (26.0%). Attack rates in asymptomatic staff (n = 70) were 4.0%, although the authors report that absence due to sickness or self-isolation were three times higher than expected. Genotyping of 19 strains showed multiple distinct clusters within 1 nursing home, and high similarity of sequences within 4 clusters [39].

An outbreak of SARS-Cov-2 across nine LTCFs in Washington, US, involving 101 residents and 50 staff members was recently described [40]. Although the attack rates of the facilities were not reported, the proportion of COVID-19 residents that required hospitalisation was high (54.5%). A separate study from one of the nine LTCFs reported an attack rate of 30% (23/76); testing was initiated after one HCW had a positive SARS-CoV-2 PCR [41]. A third report from the same outbreak estimated a doubling time that was faster in the facility (3.4 days) than what was observed in the community (5.5 days), demonstrating the potential of uncontrolled transmission in these settings [34]. In that facility, 57 of 89 residents (64%) had documented SARS-CoV-2 infection, as did 26/138 (19%) of staff members [34]. Sequencing of a convenience sample of 39 genomes from 34 residents identified 2 clusters [34].

Phylogenetic trees were produced in two of these reports, but only from patient samples and not from HCWs [34, 39]. None of these studies evaluated the transmission dynamics, in particular the direction of chains of transmission.

There are few data on risk of healthcare-associated COVID-19 in the hospital setting. One early study from China, with a sample size of 138 patients suggested that 41% of the cases were believed to be healthcare-associated [42]. Reports in the lay press suggest that in England 5–7% of all COVID-19 cases are healthcare-associated [43]. A retrospective study performed in a teaching hospital in London revealed that 15% of COVID-19 in patients between 2 March and 12 April were definitely or probably nosocomial, with a case a fatality of 36%. After the introduction of better infection control practices, the nosocomial infection rates improved.

A prospective epidemiological surveillance in a hospital trust in Cambridge combined epidemiological and genetic data to investigate causes of healthcare-associated COVID-19 [44]. The investigation revealed the existence of 35 distinct clusters, with 22% having no epidemiological evidence of transmission, and healthcare-associated clusters in 9 “non-COVID” wards involving both patients and HCWs. The results of the ongoing investigation help to adapt IPC practices, and to rule out transmission events that were initially epidemiologically suspected.

## Outbreak control strategies

Considering the high transmissibility of SARS-CoV-2 in the community (R_0_ 3.8 [2.4–5.6]) [45] and the associated high morbidity and mortality in elderly and/or comorbid patients, the preferred outbreak control strategy is to prevent its occurrence [46].

Several control measures have been implemented in the reported healthcare-associated outbreaks, although the specific effects of these measures are difficult to ascertain because they are often simultaneously implemented. Common elements include expanded testing criteria [34, 39, 41, 47–54], including testing asymptomatic patients/residents and healthcare workers, as well as serial testing or repeat point-prevalence surveys. This represents a true paradigm shift in management of nosocomial outbreaks; indeed, apart from cases where asymptomatic carriers of multidrug-resistant bacteria (e.g. methicillin-resistant *Staphylococcus aureus*) are screened [55], this practice breaks with tradition in management of respiratory viral illness. Testing is key as it has been extensively demonstrated that there are high proportions of asymptomatic, and more importantly pauci-symptomatic and pre-symptomatic individuals. Having a low threshold for testing allows for prompt identification of cases which can be managed by transferring detected patients to dedicated cohorting wards, isolation precautions, and quarantine (for HCWs and contacts). It seems suitable to perform wide-scale screening of both patients and HCWs, including asymptomatic individuals, in the event that COVID-19 cases are identified in “non-COVID” wards to help (1) identify a potential outbreak situation, and (2) being able to control it. These strategies should, preferably, be integrated into a hospital-wide surveillance system that may have variable degrees of sophistication and/or automation [56].

Accordingly, universal testing of patients upon admission has also been an innovative implemented strategy. In a Hospital in New York City, between March 22 and April 4, 2020, in a period of high community transmission of SARS-CoV-2, a universal testing strategy for all women admitted for delivery was implemented [57]. From the 215 admitted pregnant women, 4 presented symptoms and tested positive for COVID-19; from the remaining asymptomatic 211 women, 29 tested positive for SARS-CoV-2 infection; therefore, 87.9% of all the admitted pregnant women who had COVID-19, were asymptomatic at admission. However, in an area with low COVID-19 prevalence, universal screening for SARS-CoV-2 upon hospital admission revealed a small number of asymptomatic admitted patients [58].

The importance of testing of HCWs should also be underlined. As a matter of fact, in the context of outbreaks in the healthcare setting, this is a strategy that has been successfully implemented. In Minnesota, during an outbreak in a LTCF, serial testing of patients and HCWs every 7–10 days revealed to be an important infection control practice [12]. Generalized testing of HCWs has also been implemented outside the context of outbreaks in LTCF. In Egypt, HCWs were tested for SARS-CoV-2 infection either by PCR or by rapid IgM and IgG serological tests: of the 4040 screened HCWs, 170 (4.5%) were positive, with a high proportion of asymptomatic individuals [59]. A strategy of point-of-care testing, with antigen or rapid molecular testing has already been evaluated by a Cochrane Review; although it is mentioned that rapid testing may be valuable, more prospective studies in clinical settings should be performed [60].

Universal masking, including in non-clinical areas of healthcare settings, has also been commonly reported [20, 34, 41, 50, 53, 61–63], and may be useful in the context of presenteeism. Another commonly reported measure is visitor restrictions [34, 40, 41, 50, 53, 54, 61, 63, 64]. This is equally important for visitors to “non-COVID” wards (to protect the potentially uninfected patients and staff) and “COVID” wards (to prevent transmission to the potentially uninfected visitor and staff). However, pre-emptive visitor restrictions (i.e. in the absence of an outbreak) should be balanced with the mental health and quality of life of the residents [65]. Strategies to implement physical distancing inside the hospital premises, such as changing in-person meetings to virtual meetings or reorganization of the workplace can also be important measures to reduce the nosocomial transmission of SARS-CoV-2 [66].

A few Asian countries (South Korea, Taiwan, Singapore) implemented aggressive preventive strategies based on their experience with SARS-CoV-1 and MERS-CoV, combining several of the above mentioned IPC elements, mostly garnered with success in their healthcare settings [20, 64, 67]. It remains to be determined which of those elements should be routinely recommended for the 2^nd^ COVID-19 wave, to be expected in the next 12 months in most parts of the northern hemisphere.

## Expanding the research agenda

Since the beginning of the COVID-19 pandemic, numerous countries have reported variable rates of HCW infections, some of whom unfortunately succumbed to the infection. Therefore, protection of HCWs is a key priority whilst caring for COVID-19 infected patients. The debate is focused almost predominantly on the use of PPE [68], and there predominantly on selecting different types of face masks, instead of investigating other potential sources for HCW infection [69, 70]. Whilst it is clear that PPE availability is crucial, it is all the more important to ensure appropriate use of PPE, including type of PPE (e.g. surgical mask versus other), indications for PPE use; due to constraints imposed by physical distancing and work-load of IPC professionals, training may be delivered by e-learning methods [71, 72]. The effect of education and training, which can also emphasise other key concepts such as prevention of presenteeism, on nosocomial infections can be explored through web-based trials [73–75].

Given that the role of HCWs remains unclear, it is necessary to conduct studies to better distinguish community-acquired HCW infection versus patient-to-HCW transmission versus HCW-to-HCW transmission (Fig. 1). The results of these in-depth analyses will allow us to improve our understanding of SARS-CoV-2 transmission dynamics, but even more, selecting appropriate prevention measures that go beyond taking on mask types. Today, many hospitals care for COVID-19- and non-COVID-19 patients at the same time. The latter must be protected from healthcare-associated acquisition of SARS-CoV-2 during their hospital stay, and we should use the time now to anticipate better prevention strategies before the next wave of COVID-19.

As of 20 May 2020, only three studies (out of 1,621 studies on COVID-19) have been registered in the clinicaltrials.gov database with the aim of understanding nosocomial healthcare-associated transmission of SARS-CoV-2: NCT04290780, NCT04339881, and NCT04356560. From the brief summaries provided, none of these studies focus on LTCFs or geriatrics. The role of universal screening of HCWs versus targeted screening, e.g. in high-risk units (which have yet to be defined), also has a place in the research agenda.

Outbreak investigations using epidemiological techniques are the current “gold standard”, as they provide crucial spatial and temporal data on the outbreak and possible transmission routes, but also provide individual-level data such as risk factors for acquisition [76]. Nevertheless, the strengths of epidemiological outbreak investigations can be augmented by analysing genetic data. High-resolution data, such as that obtained by whole-genome sequencing, can contribute to discriminating between multiple clusters or introductions of disease within an outbreak, as well as characterizing the routes of transmission [77]. Genomic sequencing can be used to infer phylogenetic trees in order to establish contact networks that may have been unidentified by epidemiological analysis alone. However, when used alone, phylogenetic reconstruction are not without limitations, including lack of sensitivity, for example due to lower mutation rates [78, 79]. Therefore, the powerful combination of epidemiological and genetic approaches [44], which has also been previously used for other diseases, combines the advantages of each individual approach and allows the limitations of one to be compensated by the other [80, 81].

## Conclusions

The debate revolving protection against SARS-CoV-2 infection in healthcare settings has been dominated by risk of patient-to-HCW transmission and the type of PPE (N95/respirator vs. facemask) to be used for its prevention. However, it is equally important to consider protecting patients from HCW-to-patient transmission, considering the dire consequences of COVID-19. There has been a paradigm shift in outbreak management practices, that includes widespread testing of patients (or residents) and HCWs, including asymptomatic individuals. A low threshold is required in order to trigger actions to control nosocomial outbreaks and prevent further occurrences. Finally, it is crucial to deepen our understanding transmission pathways of healthcare-associated outbreaks, including the complex interplay between and respective role of HCWs and patients in transmission, in order to inform infection prevention guidelines and enhance protection of HCWs and patients.
